# Radiation-induced myocardial perfusion abnormalities in breast cancer patients following external beam radiation therapy

**Published:** 2015

**Authors:** Mohammad Eftekhari, Robabeh Anbiaei, Hanie Zamani, Babak Fallahi, Davood Beiki, Ahmad Ameri, Alireza Emami-Ardekani, Armaghan Fard-Esfahani, Ali Gholamrezanezhad, Kazem Razavi Seid Ratki, Alireza Momen Roknabadi

**Affiliations:** 1 Research Center for Nuclear Medicine, Tehran University of Medical Sciences, Tehran, Iran; 2 Department of Radiation Oncology, Imam Hossein Hospital, Shahid Beheshti University of Medical Sciences, Tehran, Iran

**Keywords:** Breast cancer, Myocardial perfusion, Radiotherapy, SPECT

## Abstract

**Objective(s):**

Radiation therapy for breast cancer can induce myocardial capillary injury and increase cardiovascular morbidity and mortality. A prospective cohort was conducted to study the prevalence of myocardial perfusion abnormalities following radiation therapy of left-sided breast cancer patients as compared to those with right–sided cancer.

**Methods:**

To minimize potential confounding factors, only those patients with low 10-year risk of coronary artery disease (based on Framingham risk scoring) were included. All patients were initially treated by modified radical mastectomy and then were managed by postoperative 3D Conformal Radiation Therapy (CRT) to the surgical bed with an additional 1-cm margin, delivered by 46-50 Gy (in 2 Gy daily fractions) over a 5-week course. The same dose-adjusted chemotherapy regimen (including anthracyclines, cyclophosphamide and taxol) was given to all patients. Six months after radiation therapy, all patients underwent cardiac SPECT for the evaluation of myocardial perfusion.

**Results:**

A total of 71 patients with a mean age of 45.3±7.2 years [35 patients with leftsided breast cancer (exposed) and 36 patients with right-sided cancer (controls)] were enrolled. Dose-volume histogram (DVH) [showing the percentage of the heart exposed to >50% of radiation] was significantly higher in patients with left-sided breast cancer. Visual interpretation detected perfusion abnormalities in 42.9% of cases and 16.7% of controls (*P*=0.02, Odds ratio=1.46). In semiquantitative segmental analysis, only apical (28.6% versus 8.3%, P=0.03) and anterolateral (17.1% versus 2.8%, P=0.049) walls showed significantly reduced myocardial perfusion in the exposed group. Summed Stress Score (SSS) of>3 was observed in twelve cases (34.3%), while in five of the controls (13.9%),(Odds ratio=1.3). There was no significant difference between the groups regarding left ventricular ejection fraction.

**Conclusion:**

The risk of radiation induced myocardial perfusion abnormality in patients treated with CRT on the left hemi thorax is not low. It is reasonable to minimize the volume of the heart being in the field of radiation employing didactic radiation planning techniques. Also it is advisable to screen these patients with MPI-SPECT, even if they are clinically asymptomatic, as early diagnosis and treatment of silent ischemia may change the outcome.

## Introduction

Breast cancer is the most common malignancy in women, accounting for approximately 26% of all female cancers (1). Surgery, chemotherapy, and radiation therapy or combinations of them are employed for treatment of this disease. Radiation therapy is often specifically used following breast conserving surgery as well as radical mastectomy of high risk patients (stages IIB, IIIA-C) (2). Postoperative radiotherapy reduces the rate of recurrence and improves the survival rate (2, 3). However, studies have shown that the therapeutic benefits from radiation therapy may be offset to some extent by both early and delayed side effects and toxicities on the heart, by this means reducing the benefits of the treatment (4).

High doses of radiation to a significant volume of myocardium can virtually injure any component of heart: Pericarditis is the classic manifestation of an acute radiation injury, while coronary artery disease (CAD), cardiomyopathy (CMP), valvular diseases, and conduction abnormalities could be seen as delayed manifestations of radiation injury, with inherent mortality and morbidity. Although previous studies suggested radiation-induced vasculopathy as the main pathophysiologic pathway responsible for cardiac side effects, the underlying mechanisms of radiation-induced CAD or CMP are still poorly understood (5). The other possible explanations are lysosomal activation, infiltration of lipid and inflammatory cell (5-7), and impaired endothelial–dependent vasodilatation (8-11).

SPECT-Gated Myocardial Perfuion Imaging (MPI) is a non-invasive procedure that can be used to detect abnormalities of perfusion and function after radiation therapy (12). Studies have shown different rates of radiation-induced perfusion abnormalities, ranging from 27 to 70% of patients being irradiated for breast cancer (1, 3, 12).

The aim of the current study was to evaluate the extent and severity of perfusion and functional abnormalities by Gated SPECT-MPI after three dimensional radiation therapy of breast cancer in patients with low 10-year risk of coronary artery disease.

## Methods

A prospective cohort study, from June 2010 to June 2012, was conducted to study the prevalence of myocardial perfusion abnormalities following radiation therapy of left-sided breast cancer patients as compared to those with right–sided cancers. A total of 71 patients with a mean age of 45.3±7.2 years (range; 32-60 years) were enrolled in the study. The patients were in stage IIB or III of a unilateral breast cancer. Thirty five patients had left-sided breast cancer (exposed group) and thirty six patients had right-sided cancer (unexposed group or controls). All patients completed the treatment protocol. They were initially treated by modified radical mastectomy or lumpectomy and then were managed by postoperative three dimensional conformal radiotherapy (3D CRT) to the surgical bed. An additional 1-cm margin was delivered by 46-50 Gy (in 2 Gy daily fractions) over a 5-week course. The patients with left- sided breast cancer were considered as exposed cases in whom a fraction of the heart was involved in the radiation field while in the patients with right-sided breast cancer (unexposed group or controls), the heart was completely out of the radiation field. To minimize potential confounding factors, only those patients without any previous history of cardiac event who had low 10-year risk of coronary artery disease (less than 5%, based on Framingham risk scoring system) receiving both postoperative 3D CRT and definitive chemotherapy were included. The point scores for gender, age, blood pressure, total cholesterol, high density lipoprotein (HDL) level and smoking were calculated and the 10-year Framingham risk score was estimated based on the total points according to standard Framingham tables. The same dose adjusted chemotherapy regimen (including anthracyclines, cyclophosphamide and taxol) was given to all patients in both groups. After obtaining informed consent, all consecutive patients who were referred to our hospital and had inclusion criteria were enrolled to the study. Exclusion criteria were considered as patients with bilateral breast cancer, history of acute coronary symptoms, pregnancy, and 10-year risk of coronary artery disease of ≥5%. The protocol was approved by the institutional review board of Tehran University of Medical Sciences.

### Imaging protocol

Six months after radiation therapy, all patients underwent cardiac SPECT for the evaluation of myocardial perfusion. A commercial sestamibi kit provided by Atomic Energy organization of Iran (AEOI) was used and the labeling and quality control procedures were performed according to the manufacturer’s instructions. Following intravenous injection of 666–814 MBq ^99m^Tc-MIBI at peak pharmacological stress with dipyridamole or dobutamine, or exercise treadmill test (ETT) at 85% of age-determined peak heart rate, all patients underwent standard stress ECG-gated SPECT. For the pharmacological stress, 0.56 mg/kg dipyridamole was infused intravenously over a 4 min period. Radiotracer was injected intravenously, 3–5 min after the completion of dipyridamole infusion. All patients were instructed to fast for at least 4 h and all beta-blocking agents, diltiazem and verapamil were discontinued 48 hours before the ETT. A rest phase study (rest ECG-gated SPECT) was performed on another day with administration of the same dose of radiotracer.

Image acquisition was performed by a rotating, single head ADAC gamma camera (Argus, ADAC, Milpitas, CA), equipped with a low energy, high resolution parallel hole, collimator with step and shoot mode, elliptical orbits, matrix size of 64 × 64 × 16, and using a roving 38.0 cm^2^ detector mask. A 20% window around the 140 keV energy peak of ^99m^Tc- was employed. Patients were lying in supine position. Thirty-two 30 sec projections were obtained over a 180° orbit (45° right anterior oblique to 45° left posterior oblique). The projection data sets were pre-filtered with a two-dimensional Butterworth filter (cut-off 0.40, order 5.0), reconstructed with filtered back-projection with no attenuation correction. The resulting transaxial image sets were reoriented into short-axis sets vertical and horizontal long-axis images for qualitative analysis.

**Figure 1:**
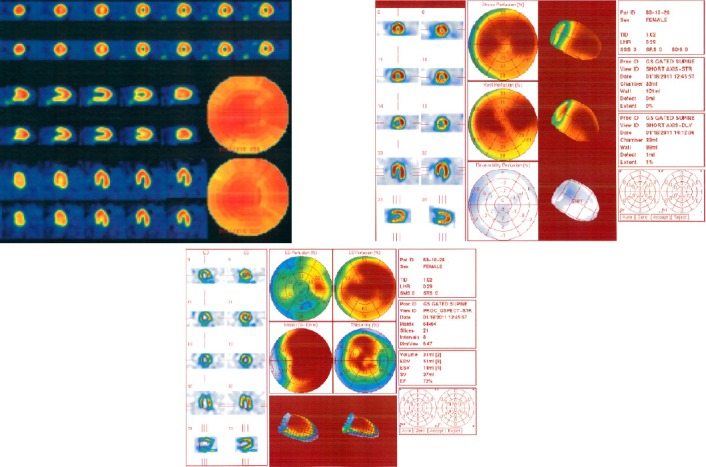
SPECT images of a patient in control group showing normal myocardial perfusion scan (1a), with QGS (1b) and QPS (1c) data. In (1a), the first and second rows are showing stress and rest phases, respectively

**Figure 2:**
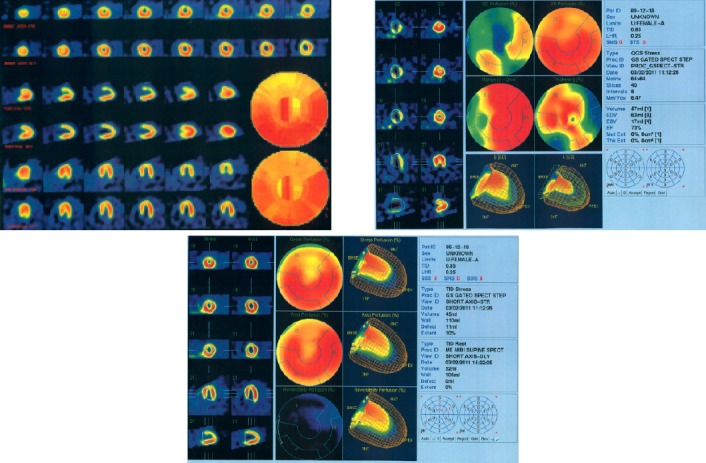
SPECT images of a patient in exposed group showing ischemia of apex, anterior and anterolateral walls (2a), with QGS (2b) and QPS (2c) data. In (2a), the first and second rows are showing stress and rest phases, respectively

The scan findings were visually interpreted by two expert nuclear medicine physicians blinded to other clinical data and angiographic results. In addition, quantitative analysis of myocardial perfusion and function was performed using the Cedars-Sinai program, according to a 20-segment model. Summed stress score (SSS), summed rest score (SRS) and summed difference score (SDS) were calculated. Also functional parameters including summed motion score (SMS) and summed thickening score (STS) as well as ejection fraction (EF) were obtained based on stress-phase gated analysis. All parameters were compared between two study groups (Figures 1, 2).

### Cumulative dose-volume histogram (DVH)

The Relative volume (%) of the heart received more than or equal to 50% of the radiation dose (DVH_50%_) was calculated based on the absolute total dose without adjustments for fraction size or overall treatment time. DVH parameters were computed from the 3D dose distributions and were exported from treatment plans. The DVH_50%_ in the exposed group was classified into 3 categories: *I*. DVH_50%_<1%, *II*. 2.5%>DVH_50%_≥1%, *III*. DVH_50%_≥2.5%.

### Statistical analysis

Differences of numerical perfusion and functional parameters for the data with normal distribution between the two groups of patients (i.e. EF) were compared by independent sample t test. Differences of categorical parameters such as semi-quantitative scores of perfusion or function (eg. SSS, SMS, …) were analyzed by Mann-Whitney U test, as the parameters did not show normal distribution. The proportional frequencies of qualitative data in the two groups were compared using Chi-square analyses. Odds ratio was also calculated for each parameter. As a multivariate analysis, a binary logistic regression test was used to calculate adjusted Odds ratios considering different interfering variables. All statistical tests were two-tailed and were performed using statistical software programs SPSS V.16.0. A P value of <0.05 was considered significant.

## Results

DVH_50%_ in the patients with left-sided breast cancer (exposed group) was 2.64±2.15 as compared to 0.0 (*P*<0.001) in the cases with right-sided breast cancer (controls). There was no significant difference between the two groups regarding patients’ age and 10-year risk of coronary artery disease (Table 1).

**Table 1:** The two groups of exposed and control patients were not significantly different regarding age and risk of coronary artery disease

	**Control**	**Exposed**	***P* Value**
Age	44.8±7.2	45.8±7.2	0.56
CAD (10 year risk)	1.8%±1.1%	2.0%±1.2%	0.48
DVH_50%_	0.0	2.6±2.2	<0.0001

CAD: Coronary Artery Disease; DVH: Dose-Volume Histogram.

Visual interpretation detected perfusion abnormalities in 15 of 35 cases (42.9%) of the exposed group and 6 of 36 (16.7%) of the controls (*P*= 0.016). The Odds ratio of perfusion abnormality in the exposed cases as compared to the controls was 3.75 (CI 95%; 1.25-11.30). In visual analysis of separate myocardial walls, only apical (28.6% versus 8.3%, *P*=0.03) and anterolateral (17.1% versus 2.8%, *P*=0.049) myocardial walls showed some degree of reversible perfusion abnormality in the exposed group, as compared to the controls (Table 2). No remarkable fixed perfusion defect was noted in each group. Semiquantitative perfusion scores confirmed the higher prevalence of myocardial perfusion abnormalities in the patients with left-sided breast cancers; Summed Stress Score (SSS) of >3 was observed in twelve exposed cases (34.3%), while five of the controls (13.9%) showing an Odds ratio of 3.24 (CI 95%; 1.01-10.47, *P*=0.044). On the other hand, only two patients (5.6%) in control group and one case (2.9%) in exposed group had SRS≥3; however, in visual assessment the rest defects in all three cases were believed to be due to attenuation artifact.

**Table 2:** Visual interpretation of myocardial perfusion study showed reversible perfusion abnormality (ischemia) in the anterolateral and apical walls, while the perfusion of the other walls was preserved. No remarkable fixed defect was visualized. The numbers indicate the number of patients with ischemia in the given myocardial wall and the rate is provided in parenthesis

**Territory of perfusion defects**	**Control** **(n: 36)**	**Exposed group (n: 35)**	***P* Value**
Anterior wall	2 (5.6%)	5 (14.13%)	0.20
Inferior wall	1 (2.8%)	1 (2.9%)	0.70
Inferoseptal wall	0	1 (2.9%)	0.50
Inferolateral wall	0	1 (2.9%)	0.50
Anteroseptal wall	2 (5.6%)	3 (8.6%)	0.50
Anterolateral wall	1(2.8%)	6 (17.1%)	0.049
Apex	3 (8.3%)	10 (28.6%)	0.03
Whole myocardium	6 (16.7%)	15 (42.9%)	0.02

Table 3 summarizes other semiquantitative perfusion and function indices of the two groups. Among all semiquantitative scores, only summed motion score was significantly reduced in patients of the exposed group. Also, there was no significant difference between the groups regarding left ventricular ejection fraction (64.5±7.0% in exposed cases versus 65.1±7.7% in controls, *P*=0.77, Odds ratio=0.06).

**Table 3:** Semiquantitative scores of myocardial perfusion and function in patients of the exposed group in comparison with the control group

Semiquantitative index	Control (n: 36)	Exposed group (n: 35)	*P* Value
SSS*	33.0 (0-13)	39.1 (0-13)	0.19
SRS*	34.5 (0-7)	37.5 (0-8)	0.29
SDS*	33.9 (0-6)	38.1(0-9)	0.35
SMS*	31.3 (0-17)	40.7 (0-15)	0.04
STS*	34.4 (0-11)	37.6 (0-11)	0.38

SSS: Summed Stress Score, SRS: Summed Rest Score, SDS: Summed Difference Score, SMS: Summed Motion Score, STS: Summed Thickening Score.

* The average score in each group is shown. The values in the parenthesis are minimum and maximum.

In multivariate logistic regression analysis, after adjusting for DVH_50%_ and age, the Odds of visual myocardial perfusion abnormality in the exposed cases was more than the controls (Odds Ratio=9.43 and CI 95% for OR=2.18 -40.75, *P*=0.01). In semiquantitative assessments, significant perfusion defects (SSS>3) were observed in 3 of 9 cases (33.3%) with less than 1% of the heart involved with equal or more than 50% of the radiation dose (DVH_50%_ <1%). In comparison, the proportional frequency of patients with SSS>3 was 40% (4 of 10) in cases with DVH_50%_ between 1 and 2.5% and 31.3% (5 of 16) in those with DVH_50%_ more than 2.5%, showing no significant relationship between perfusion defects and the percentage of the heart involved in the radiation field (*P*=0.899).

All patients in both groups were clinically followed for at least 6 months after the myocardial perfusion scan. None of the patients developed any sign or symptoms of coronary artery disease and no acute coronary event was reported.

## Discussion

A number of studies have suggested that months after radiation, serious myocardial tissue damage and perfusion abnormalities are found. Myocardial perfusion study findings of these patients can be divided into two main categories: Rest perfusion abnormalities and stress induced perfusion abnormalities. Rest perfusion abnormality, as those found by Dogan et al, indicate myocardial degeneration and fibrosis, induced directly by radiation itself, or it can also be a late complication of reduced myocardial perfusion (13). Such a degenerative fibrotic process can potentially lead to some degrees of heart failure or cardiomyopathy. Stress induced perfusion abnormalities are on the other hand, more related to vasculopathies and endothelial dysfunction induced by radiation. These reversible perfusion abnormalities pose the patient at risk of acute coronary syndrome. It is now widely appreciated that radiation induces early and late cardiac toxicities, and myocardial injury induced by radiation might lead to decreased survival due to cardiac mortality, although recurrence and cancer related death is decreased in these patients (14-16).

In our study abnormalities in perfusion were detected in 42.9% of left-sided breast cancer patients, while noted in 16.7% of right-sided breast cancer control patients, a difference which was statistically significant. Previous studies support our findings: Hardenbergh et al. (17) found that 60% of patients develop new visible perfusion defects six months after radiotherapy, but the incidence of perfusion defect in those patients treated with doxorubicin-based regimen was 70%. It seems that some chemotherapeutic agents have radiation sensitizing effects, which pose the patients at higher risk of radiation induced vasculopathy and myocardial perfusion defects. Such a hypothesis needs to be studied in larger samples of patients. In a prospective study by Gyenes et al. (12), 50% of breast cancer patients treated with radiation to the left hemithorax developed new myocardial perfusion defects one year following radiotherapy.

Apex is the most common segment affected by radiation. In our study perfusion abnormalities were confined to the apex in 28.6% of cases, which is in accordance with what Seddon et al. reported (3). Limited vascularity and watershed perfusion of apex, makes it susceptible to any kind of ischemic events, e.g. ischemia induced by atherosclerotic or radiation induced vasculopathies. This is why the prevalence of cardiac aneurysms is highest at the apex. In our study, SMS of the cases was significantly higher than the controls. The impaired motion and function of the involved segments could be explained by radiation-induced perfusion abnormality leading to a stunning phenomenon, or direct radiation toxicity to myocytes resulting in a chronic fibrotic process; however, the exact underlying mechanism needs further investigation by modalities like magnetic resonance imaging or pathologic assessment.

In our study as well as and Seddon’s report (3), 16.7% of right sided breast cancer patients suffered from some degrees of myocardial perfusion defect. These perfusion abnormalities could be explained by the back scatter of radiation or the effect of adjunctive chemotherapy; however, the underlying mechanism is still to be investigated. Two other studies by Tishler et al. (18) and Chakravarthy et al. (19) also showed that chemotherapy with 5-FU, Taxol and Herceptin are associated with cardiac toxicities secondary to their radiation sensitizing effect, or even irrespective of the role of radiation. As all our patients were treated by Taxol, some degrees of myocardial perfusion abnormality in the controls could be expected.

Although we did not find any relationship between myocardial perfusion abnormalities and DVH_50%_, Mark et al. reported that the incidence of perfusion defects is related to the volume of the heart included within the radiation field (20). We believe that longer follow up and larger sample sizes could unmask such an association. As in accordance with Mark et al. (20), we did not find any relationship between the extent and severity of regional perfusion abnormalities and LVEF changes. This can be explained by the findings of Borges-Neto et al. (21), who demonstrated that significant reduction in LVEF is not detected until >30% of LV suffers from perfusion abnormality. Both in the Mark’s study and ours, the percentage of LV involved in the radiation field was less than 10%, so significant drop in LVEF is not expected.

### Study limitations

The main limitation of our study was the lack of baseline myocardial perfusion study. However, as the 10 year risk of coronary event in our population was less than 5%, we could assume the baseline SPECT to be normal.

Also, six month-period is rather a short term of follow up for radiation induced coronary symptoms in irradiated patients. This is probably why all our patients were clinically asymptomatic, while had evidences of inducible ischemia scintigraphically. It is likely that they might become symptomatic in longer follow ups. The retrospective case-control study by Seddon et al. (3) is in favor of such a conclusion: myocardial perfusion abnormalities were noted in 70.8% of left-sided patients and 16.7% of right-sided breast cancers, while the corresponding rates in our study were 42.9% and 16.7%. The longer follow up of patients in Seddon’s study (5 years versus 6 months in our study) can explain the higher detection rate of myocardial perfusion abnormalities. In another study by Mark et al. (20) myocardial perfusion defects were noted in 27%, 29%, 38% and 42% of patients at 6, 12, 18 and 24 months after radiotherapy, respectively, a fact which confirms that the development of myocardial perfusion defects is related to the time interval following radiation therapy. Other studies have shown that the mean time interval for development of radiation induced CAD is approximately 82 months (59-104 months) (22).

In conclusion, the risk of radiation induced myocardial perfusion abnormality in patients treated with CRT on the left hemithorax is not low. It is reasonable to minimize the volume of the heart being in the field of radiation employing didactic radiation planning techniques. Also it is advisable to screen these patients with MPI-SPECT, even if they are clinically asymptomatic, as early diagnosis and treatment of silent ischemia could change the outcome.
